# Analysis of Prevalence and Clinical Features of Aortic Stenosis in Patients with and without Bicuspid Aortic Valve Using Machine Learning Methods

**DOI:** 10.3390/jpm13111588

**Published:** 2023-11-09

**Authors:** Olga Irtyuga, Mary Babakekhyan, Anna Kostareva, Vladimir Uspensky, Michail Gordeev, Giuseppe Faggian, Anna Malashicheva, Oleg Metsker, Evgeny Shlyakhto, Georgy Kopanitsa

**Affiliations:** 1 Almazov National Medical Research Centre, 197341 Saint-Petersburg, Russia; irtyuga_ob@almazovcentre.ru (O.I.); babakekhyan_mv@almazovcentre.ru (M.B.); kostareva_aa@almazovcentre.ru (A.K.); uspenskiy_ve@almazovcentre.ru (V.U.); gordeev_ml@almazovcentre.ru (M.G.); malashicheva_ab@almazovcentre.ru (A.M.); metsker_og@almazovcentre.ru (O.M.); shlyakhto_ev@almazovcentre.ru (E.S.); 2 Department of Cardiac Surgery, University of Verona Medical School, 37134 Verona, Italy; giuseppe.faggian@univr.it

**Keywords:** machine learning, aortic stenosis, predictors, retrospective study, clinical features

## Abstract

Aortic stenosis (AS) is the most commonly diagnosed valvular heart disease, and its prevalence increases with the aging of the general population. However, AS is often diagnosed at a severe stage, necessitating surgical treatment, due to its long asymptomatic period. The objective of this study was to analyze the frequency of AS in a population of cardiovascular patients using echocardiography (ECHO) and to identify clinical factors and features associated with these patient groups. We utilized machine learning methods to analyze 84,851 echocardiograms performed between 2010 and 2018 at the National Medical Research Center named after V.A. Almazov. The primary indications for ECHO were coronary artery disease (CAD) and hypertension (HP), accounting for 33.5% and 14.2% of the cases, respectively. The frequency of AS was found to be 13.26% among the patients (n = 11,252). Within our study, 1544 patients had a bicuspid aortic valve (BAV), while 83,316 patients had a tricuspid aortic valve (TAV). BAV patients were observed to be younger compared to TAV patients. AS was more prevalent in the BAV group (59%) compared to the TAV group (12%), with a *p*-value of <0.0001. By employing a machine learning algorithm, we randomly identified significant features present in AS patients, including age, hypertension (HP), aortic regurgitation (AR), ascending aortic dilatation (AscAD), and BAV. These findings could serve as additional indications for earlier observation and more frequent ECHO in specific patient groups for the earlier detection of developing AS.

## 1. Introduction

This paper is an extension of the paper presented in the pHealth 2021 Conference [1]. AS is the most common acquired valvular heart disease [2,3,4,5], and the prevalence of moderate or severe valvular disease increases with age, which poses a burden on healthcare systems in developed countries [2,3,4]. Bicuspid aortic valve (BAV) holds a significant position within the population affected by aortic stenosis [6,7], and it is recognized as one of the most prevalent congenital heart defects [8]. The detectability of BAV in the general population varies between 0.5% and 2%, often associated with aortic pathology [8,9,10]. Recent studies have revealed a lower diagnosis rate of AS in women compared to men, suggesting an important imbalance given the associated lower survival rates [11]. Further investigations into sex-related differences in AS, regardless of its severity, are warranted [12,13].

The formation of aortic stenosis remains a topic without a unified consensus. The question of early diagnosis and its impact on disease progression, aiming to reduce the need for surgical treatment, remains unanswered. Our knowledge regarding predictors of symptom development and adverse outcomes primarily focuses on data concerning asymptomatic severe AS, with a lack of information regarding the natural history of AS. Numerous studies aim to identify factors influencing the rate, timing, and extent of aortic stenosis, regardless of its morphology [4,14]. This field of research may help refine the indications for earlier echocardiographic screening in specific populations. Currently, echocardiography is recommended for patients with an unexplained systolic noise on the aortic valve, second heart sound, a history of BAV, or symptoms that may be attributed to AS [7]. Few studies have explored the role of risk factors in the onset and progression of AS, with functional status being one of the few indicators investigated [13]. No significant differences have been observed for age, sex, cause of aortic stenosis, comorbid diseases, smoking history, or coexisting coronary artery disease (CAD). Some population-based studies have indicated that higher body mass index and obesity correlate with the occurrence of aortic stenosis, although these findings are specific to certain races and geographical locations [15,16]. Limited data are available regarding predictors of AS development in mild and moderate cases, although indicators such as the degree of aortic valve calcification and the presence of CAD have been associated with progression and prognosis [14,17].

Echocardiography is the key diagnostic method for AS [3,6], and there are currently no medical therapies known to influence the natural history of aortic stenosis, as per the recent ESC guidelines [3]. Large medical centers have the capacity to collect, store, and analyze substantial amounts of information [18]. This wealth of data can be utilized for statistical analysis and machine learning methods to uncover new relationships between risk factors and the development of asymptomatic diseases. Consequently, our study enables the evaluation of a specific population’s prevalence of AS and its associations with various predictors.

The goal of this study is to identify the most important risk factors in the development of aortic stenosis, focusing on their prognostic significance. To attain this objective, we thoroughly analyze the importance of various predictors and distinguish between risk factors associated with aortic stenosis and those that predict the occurrence of ascending aortic aneurysms.

## 2. Materials and Methods

The research plan received authorization from the ethics committee at the Almazov National Medical Research Centre in Saint Petersburg, Russian Federation, following the guidelines specified in the Declaration of Helsinki before the study was initiated.

### 2.1. Study Cohort

To identify patients with aortic stenosis (AS) and different valve morphologies (bicuspid aortic valve—BAV, and tricuspid aortic valve—TAV), we conducted a retrospective analysis of the ECHO database at the Almazov National Medical Research Centre. The database encompassed 145,454 echocardiograms from both outpatients and hospitalized patients who received observation and treatment at the center between January 2010 and November 2018. It is important to note that the patient cohort used in this analysis is the same as the one utilized in our previous study [1]. Please see Table 1.

**Figure 1:**
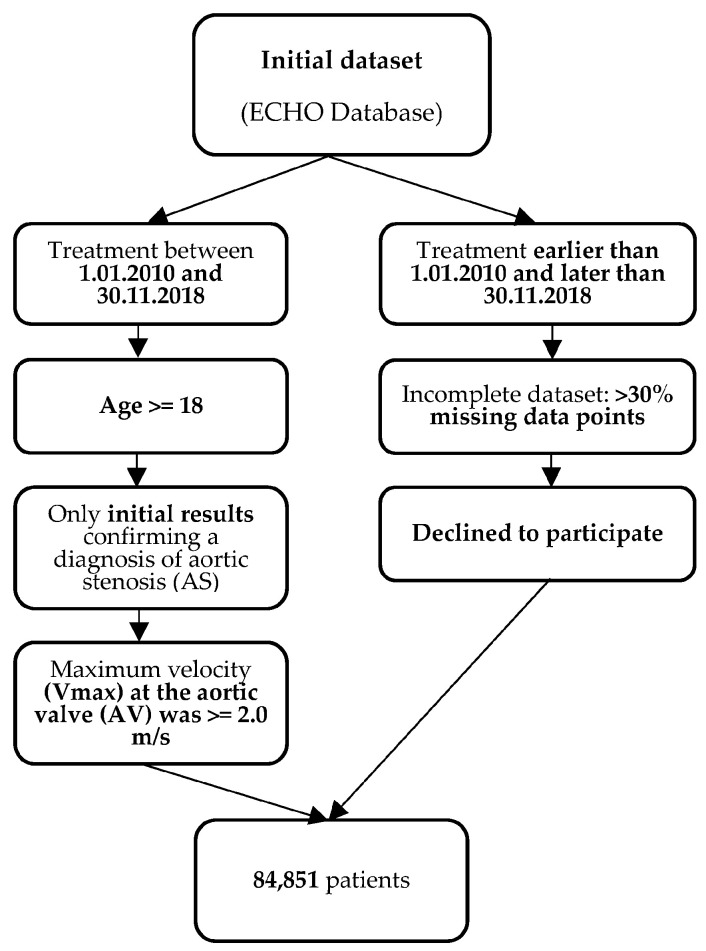
Inclusion and exclusion criteria.

We used the following criteria in the process of data collection (Figure 1):

#### 2.1.1. Inclusion Criteria

1. Patients from the ECHO database who initiated treatment between 1 January 2010 and 30 November 2018;
2. For patients who underwent multiple ECHO examinations during this period, only the initial results confirming a diagnosis of aortic stenosis (AS) were considered for the study. ECHO examinations were primarily conducted in the following clinical scenarios: suspected cardiac etiology based on symptoms, signs, or other tests, as well as evaluation and follow-up of individuals with cardiovascular disease;
3. The age of the patients was equal to or greater than 18 years;
4. Patients were included in the study if the maximum velocity (Vmax) at the aortic valve (AV) was equal to or greater than 2.0 m/s, based on the definition of AS outlined in the 2020 ACC/AHA Guideline for the Management of Patients with Valvular Heart Disease [6].

#### 2.1.2. Exclusion Criteria

1. Patients whose treatment started before 1 January 2010 or ended after 30 November 2018;
2. Patients who had incomplete datasets;
3. Patients who declined to participate in the study.

To confirm AS we also considered the mean aortic transvalvular pressure gradient and aortic valve area. The AV velocity served as the cutoff point for further analysis. Additionally, the average gradient on the AV and the size of the AV orifice were considered. However, it is worth noting that not all conclusions included these features. The presence of AS was confirmed through its mentioning in the electronic medical record diagnosis and was defined using the International Classification of Diseases, 10th Revision, Clinical Modification (ICD-10-CM) codes I35.0 (Aortic (valve) stenosis), I35.1 (Nonrheumatic aortic (valve) insufficiency), I35.2 (Aortic (valve) stenosis with insufficiency), I35.8 (Other nonrheumatic aortic valve disorders), and I35.9 (Nonrheumatic aortic valve disorder, unspecified).

The initial dataset contained 51 features, but for 25 of them, we had less than 30% of data points available. After removing these features, we ended with a dataset with 26 features. The resulting dataset contained 26 predictors: age, gender, aorta maximum diameter (max aorta), aortic diameter at the sinus of the Valsalva (max aorta sinus), systolic blood pressure (SPB), diastolic blood pressure (DBP), pulse blood pressure (SBP), AR, BAV, HP, CAD, heart failure, congenital heart disease (CHD), atrial fibrillation (AF), thoracic aortic aneurysm (TAO), hyperlipidemia, diabetes mellitus (DM), obesity, ulcer disease, chronic obstructive pulmonary disease (COPD), cholecystitis, stroke, asthma, cholelithiasis, goiter, and thyroid disorders. All drugs taken by patients were combined into two groups: antihypertensive and lipid-lowering.

A retrospective analysis was conducted on a total of 84,851 cases that satisfied the predetermined inclusion and exclusion criteria. All patients were categorized into two subgroups: those with tricuspid aortic valve (TAV) (n = 83,316) and those with bicuspid aortic valve (BAV) (n = 1544).

All patients underwent comprehensive two-dimensional and Doppler transthoracic echocardiography, following the latest echocardiography guidelines. The Vivid 7.0 system (GE, Philadelphia, PA, USA) was employed for the echocardiographic examinations [6,19].

Aortic diameters, ventricular sizes, ventricular function, and valve performance measurements were conducted in accordance with the current recommendations for echocardiography [6,19]. The maximal aortic diameter was indexed to the body surface area to obtain an absolute value.

The diagnosis of bicuspid aortic valve (BAV) was established through short-axis imaging of the aortic valve (AV), which revealed the presence of only two commissures, delineating two AV cusps.

Additionally, for each echocardiography case, an analysis was performed to determine the reason for ordering the echocardiogram. In 33.5% of cases, echocardiography was performed for patients with coronary artery disease (CAD), while in 14.2% of cases, it was conducted for patients with hypertension (HP).

Table 1 and Table 2 present the clinical and demographic characteristics of the patient groups, respectively.

### 2.2. Statistical Methods

The statistical analysis was conducted using STATISTICA v. 10.0 (StatSoft Inc., Tulsa, OK, USA). The baseline characteristics of the study population were presented as percentages for qualitative variables and as medians and quartiles for quantitative variables that were not normally distributed, as deemed appropriate. The *p*-test was utilized to determine the probability of the distribution of characteristic values between different groups of patients with bicuspid aortic valve (BAV) compared to those with tricuspid aortic valve (TAV). Notably, due to the significant differences observed between sexes in terms of demographic characteristics, all analyses were separately reported for men and women.

### 2.3. Data Preprocessing

To filter out obvious outliers, we eliminated 1% of values with the highest *z*-scores. Subsequently, we applied min–max normalization to the remaining values.

### 2.4. Classification Model Grid Search and Features Importance

To determine the best performance model among Artificial Neural Network (ANN), Support Vector Machine (SVM), Decision Tree (DT), and Random Forest (RF), each experiment ran in the setting of stratified 5-fold cross-validation, i.e., a random 80% of the training dataset was used for training and a random 20% of the training dataset for testing. Target class ratios in the folds were preserved. For the performance assessment of SVM and DT classifiers, we ran it 100 times; 100 × 5-fold cross-validation resulted in 500 predictions. All the measurements were performed separately per dataset and per model parameter value to determine the best parameters for classifiers as well as optimal data preprocessing. After determining the optimal dataset and model parameters, we performed a validation with the testing dataset. As an additional performance assessment score, we used the AUC of the ROC, which represents the trade-off between the sensitivity and specificity of the model. We used a series of classification models available within scikit-learn as a pool for the selection of the best predictive methods to be applied within the proposed scheme. A summary of the required model parameters is presented in Table 3. The algorithm was implemented in Python 3.6.3 with the scikit-learn 0.19.1 library (https://scikit-learn.org/stable/ accessed on 20 October 2023).

A Random Forest (RF) is an ensemble of machine-learning algorithms that combines multiple tree predictors. Each tree in the forest relies on values from a randomly sampled vector, independently and with the same distribution for all trees.

Feature importance measurements were performed with the RF model as the best-performing model for our settings.

The *p*-value was calculated using different methods. For categorical features, the chi-square criterion was applied, while for continuous features, the Kolmogorov-Smirnov test was utilized.

Python 3 packages, including scikit-learn [20] and Catboost [21], were employed for implementing machine-learning models, seaborn [22] and matplotlib [23] for data visualization, SMOTE [24] for dataset balancing, and SHapley Additive exPlanations (SHAP) [25] for interpreting black-box results. The discrimination of the models was evaluated using ROC curves.

The Shapley value for each feature was calculated as the average contribution it makes across all possible combinations of features, with different permutations of features getting equal weight. Shapley values provide a way to fairly distribute the value (in this case, the prediction) among the features in a predictive model. Shapley values offer a more comprehensive and fair understanding of how each feature contributes to the model’s predictions.

## 3. Results

**Figure 2:**
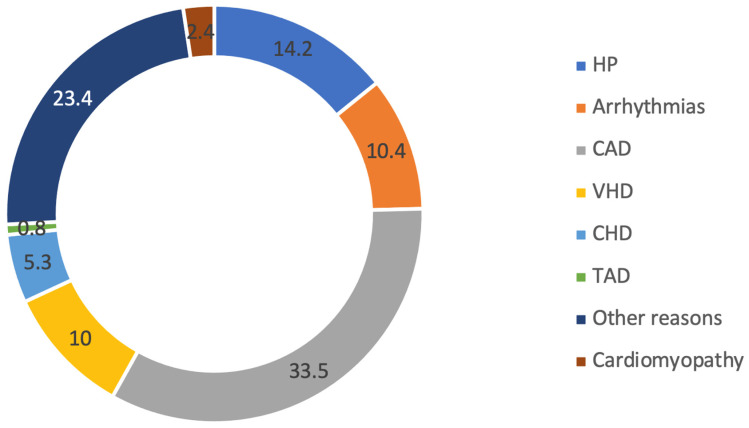
Study population characteristics.

The study included a population of 84,851 patients who underwent screening by echocardiography (ECHO). The primary indications for performing ECHO included the presence of coronary artery disease (CAD), hypertension (HP), known valvular heart disease (VHD), various forms of arrhythmia, as well as other reasons (refer to Figure 2 for details). The article on the clinical characteristics of ascending aortic dilatation in patients with and without bicuspid aortic valve (BAV) [16] provides a comprehensive description of the patients’ referral reasons.

BAV was detected in 1544 patients, according to ECHO, while TAV was detected in 83,316 patients. AS was diagnosed in 11,252 (13.26%) patients. At the same time AS was more frequent in the group of patients with BAV (n = 901, 58.7%): 59.5% (n = 536) in men and 40.5% (n = 365) in women than in the group of TAV (n = 10,351, 12.42%): 42.7% (n = 4423) in men and 57.3% (n = 5928) in women (*p* < 0.0001).

The results showed that both male and female patients with AS were significantly older, had heart failure more often, and had wider diameter of ascending aorta and higher Vmax, regardless of AV morphology (Table 1, *p* < 0.0001). Patients with AS and BAV had hyperlipidemia, CAD, COPD, increased weight, and obesity more often in comparison to patients without AS (*p* < 0.01). Higher blood pressure (both systolic and diastolic blood pressure) was registered in the TAV group. Patients without BAV but with AS had diagnoses of aortic regurgitation more often (*p* < 0.0001). Also, female patients without BAV but with AS had more common diagnoses of hyperlipidemia, CAD, and obesity (*p* < 0.01) (Table 1 and Table 2).

The grid search results are presented in Table 3 with Random Forest showing the best overall performance.

**Figure 3:**
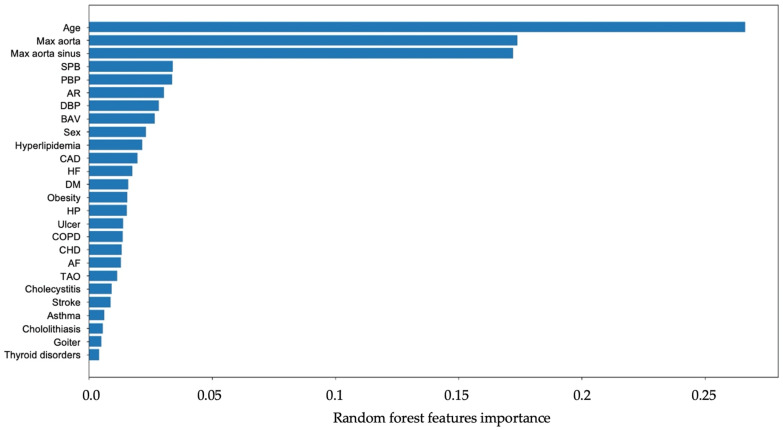
The results of the analysis of features important to the development of AS.

**Figure 4:**
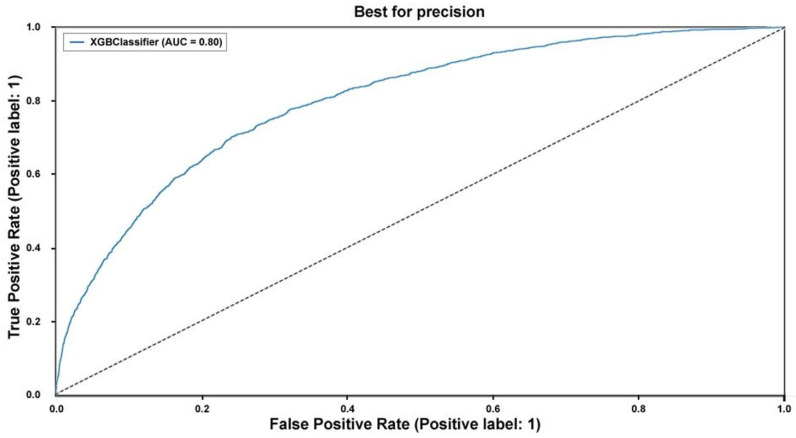
ROC for the classification model for the presence of AS.

In AS patients, diabetes mellitus, obesity, ulcers, COPD, and cholecystitis appeared as less important predictors (Figure 3). The area under the curve (AUC) of the receiver operating characteristic (ROC) was calculated to be 0.80. The corresponding ROC curve is depicted in Figure 4.

**Figure 5:**
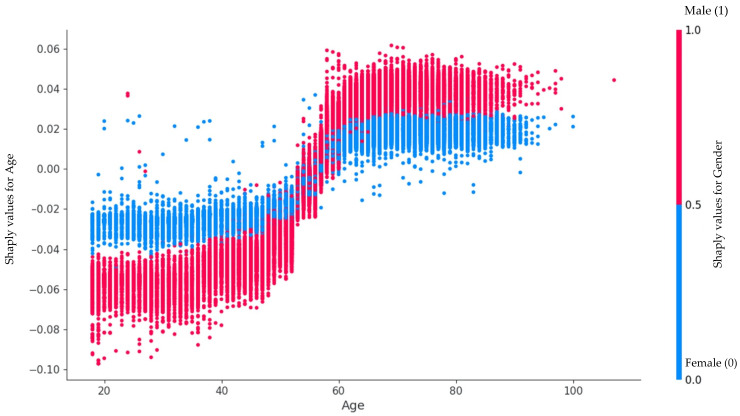
Influence of age in relation to gender on the formation of AS.

Figure 5 shows that gender changes its influence on the AS at 60. Women younger than 60 have higher chances of getting AS than men of the same age. This reverses after a cross point at 60 years old.

**Figure 6:**
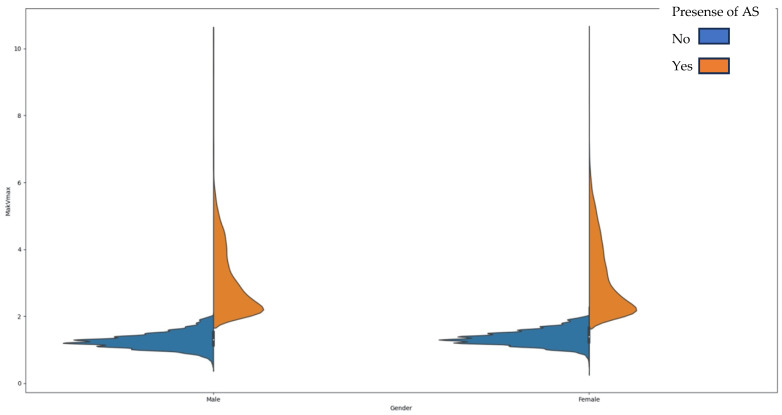
Distribution of the severity of AS depending on age and gender.

Figure 6 reveals an age-related difference in how Aortic Stenosis develops in men and women. It is clear that AS tends to appear at a younger age in women, while for men, it becomes significant at an older age. What is particularly interesting is that this shift occurs right around the age of 60, marking a turning point in the way age impacts AS formation in both genders.

## 4. Discussion

The objective of our study was to conduct a comprehensive assessment of predictors and the prognostic significance of various factors on the prevalence of AS. As anticipated, age was identified as the primary factor associated with AS formation, aligning with previously reported findings. Additionally, aortic dilatation, hypertension, congenital heart defects, bicuspid aortic valve (BAV), and gender were also regarded as significant factors in our analysis.

### 4.1. Features Importance

We used a machine learning approach with Random Forest to determine the five most significant features for patients with AS. Two of them, namely older age and hypertension, are similar to the AscAD population in our previous publication [1]. The presence of BAV and dyslipidemia in AS patients was more significant than in AscAD patients. Moreover, important features of AS formation included an increase in the size of the ascending aorta. On the contrary, male sex was less important than in AscAD patients.

The best-performing model in the study was a Random Forest model. All feature importance measurements were made using this model. The application of Random Forest for this type of task can have the following benefits. Random Forest is less sensitive to outliers because it relies on the majority vote of multiple Decision Trees. Conventional statistical methods, like linear regression, can be heavily influenced by outliers. Random Forest provides a measure of feature importance, which can be valuable for variable selection and understanding which features contribute most to the prediction. Random Forest does not require assumptions about the distribution of the data, making them more versatile for analyzing various types of data, including both numerical and categorical variables. Random Forest can handle missing data without the need for imputation or specialized techniques.

In recent years, the differences in the course of AS depending on gender have been mentioned more and more often [11,26,27]. In our work, we also obtained significant differences between the groups, so in the following, we analyzed them separately.

Additionally, it was observed that women with AS (aortic stenosis) outnumbered men by more than 1000, despite the frequency of echoes being equal in both groups. Traditionally, AS has been believed to be 2–3 times more common in men [28]. However, recent research by Ana C. Iribarren et al. and Toyofuku Ms. et al. has challenged the notion of AS prevalence being dependent on gender. These studies showed that women with AS tend to be older and more common among patients over 75 years of age. We identified that gender changes its influence on AS at the age of 60. Women under 60 have a higher chance of developing AS than men of the same age. This changes after the crossover point at age 60 [12].

The hypothesis put forward was that women are more likely to seek medical care, including preventive care, which led to the assumption that women would have milder cases of AS. To investigate this hypothesis, additional analysis was conducted. Surprisingly, the results contradicted the hypothesis. The number of patients with mild and moderate stenosis did not differ significantly between men and women. However, a clear prevalence of women with severe stenosis was observed [29]. Ana C. Iribarren et al. demonstrated that due to physiological differences, severe AS is diagnosed later in women, resulting in delayed medical care-seeking [11,30]. The findings of this study suggested that men with severe AS may not seek medical care due to shorter life expectancy compared to women and other causes of death.

In our study, BAV was detected in 1544 patients, while TAV was detected in 83,316 patients. Patients with BAV were younger than TAV patients, as in the population. AS was diagnosed in 11,252 (13.26%) patients. AS was more frequent in the group of patients with BAV (58.77%) than in the group of TAV (12.4%).

The study included patients according to the ACC/AHA Guideline for the Management of Patients With Valvular Heart Disease [6], characterizing the presence of aortic stenosis (AS) when the aortic valve velocity was above 2 m/s. Out of 4432 patients, the median value of the ascending aorta diameter was 37 (33; 40) mm, and in the area of the sinuses of Valsalva, it was 36 (33; 39) mm. Aortic regurgitation of varying degrees of severity, predominantly mild, was observed in 17% of patients. Thus, we believe that normal aortic values and mild aortic regurgitation in the vast majority of included patients could not have influenced the aortic valve velocity measurements and the results of the analysis.

The findings from the study confirmed that age and bicuspid aortic valve are significant risk factors for AS. However, it was unexpected to discover that male sex was not among the top significant risk factors. Instead, aortic dilatation, aortic regurgitation (AR), and hypertension (HP) were found to be more prominent risk factors. This finding was consistent with the research of Shen M. et al. and Généreux P. et al., who demonstrated that the presence of AR, HP, and DM in patients with BAV and mild/moderate AS led to faster hemodynamic and anatomical progression of AS [31,32]. Upon analyzing clinical characteristics, it was observed that patients with BAV had AS recorded at an average age of around 50 years, whereas patients with Tricuspid Aortic Valve (TAV) were mostly diagnosed after the age of 60. Nevertheless, both groups of patients were notably older than those without AS, aligning with global data [33]. The study results showed that hypertension (HP) was present in 56% of patients with CHD:BAV and 71% of patients with TAV. According to the current analysis, the diameter of the ascending aorta was larger in the group of patients with AS and BAV compared to patients with normal valve morphology, which was in line with the results of other studies [34]. Additionally, aortic regurgitation (AR) was identified as a predictor of AS regardless of valve morphology, and varying degrees of AR was detected in one in four patients with BAV. Other studies have also reported the incidence of AR in patients with BAV to range from 13 to 65% [34,35]. Statistically, the median ejection fraction (EF) was within the normal range. However, it is worth mentioning that this median data were derived from a sample size of over 80,000 individuals. The quartiles indicate that EF levels below 55% were observed in all groups, and some patients were diagnosed with HF (heart failure) with preserved EF, considering the clinical context as well. To summarize, there were 35,192 patients diagnosed with HF, which is higher than the average frequency of occurrence. However, considering that it is a cardiology clinic with a specialized center for the treatment of HF, such a number of cases is expected [36].

Currently, echocardiography (ECHO) is recommended for patients with unexplained systolic noise on the aortic valve of the heart, second heart sound, a history of bicuspid aortic valve (BAV), or symptoms that could be attributed to AS [30]. However, this may not be sufficient considering the known information about delayed diagnosis of asymptomatic AS, including severe cases and instances of rapid AS progression, which are not uncommon [37]. In fact, AS has been accidentally diagnosed during examinations for other diseases, such as coronary artery disease (CAD), hypertension (HP), known valve heart disease (VHD), various arrhythmias, and other reasons. The optimal timing for follow-up examinations in cases of mild and moderate AS is still unknown [6], and the appropriate timing for the first ECHO in specific groups of asymptomatic individuals remains a question. We believe that men over the age of 60 should have a screening ECHO. These issues also pose logistical and economic challenges.

Therefore, it would be desirable to identify predictors of outcomes that enable risk assessment and more personalized management strategies.

Using the machine learning algorithm, we randomly identified significant features occurring in AS patients: age, HP, AR, AscAD, and BAV. Our findings could serve as additional indications for earlier observation and ECHO in specific patient groups. We think that it is necessary to perform ECHO more frequently to prevent the formation of AS and timely identify the indications for surgical treatment.

### 4.2. Study Limitations

The first limitation of this study is its retrospective nature and reliance on single-center data. Only one echocardiogram was analyzed in each case, so there was no assessment of AS progression within this population. The second limitation pertains to the referral nature of the center. The patients were not randomly selected from the general population but rather underwent examinations in a medical research center due to clinical indications and comorbidities. This fact may introduce some selection bias since this particular population of patients carries a higher risk.

In the confirmation of AS, we considered the mean aortic transvalvular pressure gradient and aortic valve area. However, it is important to note that these features were not present in all ECHO findings, which is a limitation of the current study. In addition to the ECHO features, we also used the ICD-10 codes I35.0, I35.1, I35.2, I35.8, and I35.9 for the confirmation of AS.

Machine learning analysis possesses inherent limitations associated with the availability and occupancy of the medical database. However, due to the utilization of a substantial dataset, these drawbacks are balanced out to a great extent.

Future research efforts should focus on early diagnostics and strategies to delay the progression of degenerative aortic valve disease by examining a more representative sample from the general population.

## 5. Conclusions

AS, one of the most common acquired valve heart diseases [2,3], was detected in 13.26% (11,252) of the population analyzed in this study. Therefore, the objective of our study was to identify significant factors and clinical conditions in patients with AS. Through the implementation of a machine learning algorithm, we randomly identified the following significant features in AS patients: age, hypertension (HP), aortic regurgitation (AR), ascending aortic dilatation (AscAD), and BAV. These findings highlight the main factors associated with AS. These findings could serve as additional indications for earlier observation and more frequent ECHO in specific patient groups for the earlier detection of developing AS.

## Figures and Tables

**Table 1:** Clinical and demographic characteristics of male patients.

Features	BAV, n = 983 Median; Quartiles			TAV, n = 39,703 Median; Quartiles		
	With AS, n = 536	Without AS, n = 447	*p*	With AS, n = 4423	Without AS, n = 35,280	*p*
Age, years (median and bounds)	50 (34; 60)	29 (21; 46)	<0.0001	66 (57; 74)	57 (46; 65)	<0.0001
Aortic diameter at the sinus of the Valsalva, mm	37 (34; 41)	37 (33; 41)	<0.05	36 (34; 39)	36 (33; 39)	<0.0001
Aortic diameter at the proximal ascending aorta, mm	39 (35; 44)	24.8 (21.8; 27.8)	<0.0001	37 (34; 40)	34 (31; 37)	<0.0001
BMI, kg/m^2^	26.3 (23.9; 30)	24.8 (21.8; 27.8)	<0.0001	27.3 (24.5; 30.3)	27.4 (24.5; 30.6)	0.48
AS dpmax, mmHg	29 (20; 50)	10 (7; 12)	<0.0001	30 (20; 53)	6 (5; 8)	<0.0001
EF LV (%)	63.8 (57.4; 69)	64.2 (59.5; 69.7)	0.04	62.4 (53.4; 68)	60.9 (51.5; 67)	<0.0001
SBP office, mmHg	135 (124; 142)	130 (120; 140)	0.12	140 (129; 150)	130 (120; 140)	<0.0001
DBP office, mmHg	80 (75; 85)	80 (80; 85)	0.86	80 (80; 88)	80 (80; 87)	0.46
AR, n (%)	123 (22.95)	118 (26.46)	0.20	778 (17.59)	1286 (3.65)	<0.0001
Hypertension, n (%)	316 (58.95)	268 (59.96)	0. 5	2803 (63.37)	25,532 (72.37)	<0.001
Diabetes mellitus, n (%)	31 (5.78)	17 (3.80)	0.15	451 (10.20)	3204 (9.08)	0. 2
CAD, n (%)	127 (23.69)	46 (10.29)	<0.001	1818 (41.10)	14,222 (40.31)	0.31
COPD, n (%)	58 (10.82)	23 (5.15)	0.001	460 (10.40)	3687 (10.45)	0.92
Asthma, n (%)	22 (4.10)	9 (2.01)	0.06	88 (1.99)	732(2.07)	0.71
Obesity, (BMI > 30), n (%)	61 (11.3)	19 (4.25)	<0.0001	394 (8.9)	3005 (8.52)	0.15
Hyperlipidemia, n (%)	135 (25.19)	56 (12.53)	<0.0001	1170 (26.45)	9061 (25.68)	0.27
Heart failure, n (%)	320 (59.70)	156 (34.90)	<0.0001	2332 (52.72)	14,770 (41.87)	<0.001

BMI—body mass index; SBP—systolic blood pressure; DBP—diastolic blood pressure; AS dpmax—antegrade gradient across the narrowed aortic valve; EF LV—left ventricular ejection fraction, AR—aortic regurgitation; COPD—chronic obstructive pulmonary disease; CAD—coronary artery disease.

**Table 2:** Clinical and demographic characteristics of female patients.

Variables	BAV, n = 541 Median; Quartiles			TAV, n = 43,613 Median; Quartiles		
	With AS, n = 365	Without AS, n = 185	*p*	With AS, n = 5928	Without AS, n = 40,925	*p*
Age, years (median and bounds)	49 (31; 61)	31 (26; 49);	<0.0001	71 (62; 77)	58 (41; 68);	<0.0001
Aortic diameter at the sinus of the Valsalva, mm	32 (30; 35)	32 (29; 36)	0.89	32 (30; 35)	32 (30; 34)	<0.0001
Aortic diameter at the proximal ascending aorta, mm	36 (32; 40)	33 (29; 39)	<0.0001	34 (31; 37)	31 (28; 34)	<0.0001
BMI, kg/m^2^	25.6 (22.6; 29.9)	24.6 (21.7; 26.9)	0.01	28.8 (25.2; 32.6)	27.1 (23.5; 31.2)	<0.0001
AS dpmax, mmHg	32 (22; 56)	10 (8; 13)	<0.0001	31 (20; 60)	7 (5; 9)	<0.0001
EF LV (%)	66.9 (61.8; 71.4)	66 (61; 70)	0.15	65.9 (60.7; 70)	65.7 (60.6; 70)	0.34
SBP office, mmHg	120 (120; 140)	120 (110; 127.5)	0.13	140 (130; 150)	130 (120; 140)	<0.0001
DBP office, mmHg	80 (70; 80)	80 (70; 80)	0.61	80 (80; 90)	80 (75; 85)	<0.0001
AR, n (%)	64 (17.53)	27 (14.59)	0.38	814 (13.74)	1284 (3.14)	<0.0001
Hypertension, n (%)	177 (48.5)	99 (53.5)	0. 1	3589 (60.5)	26,923 (65.78)	0.3
Diabetes mellitus, n (%)	20 (5.48)	9 (4.86)	0.76	824 (13.9)	3870 (9.45)	0.1
CAD, n (%)	58 (15.89)	18 (9.73)	0.05	2183 (36.83)	9968 (26.45)	<0.0001
COPD, n (%)	15 (4.11)	5 (2.70)	0.40	426 (7.19)	2144 (5.69)	0.5
Asthma, n (%)	10 (2.74)	5 (2.70)	0.98	208 (3.51)	1133 (3.01)	0.8
Obesity, (BMI > 30), n (%)	47 (12.8)	5 (2.70)	0.0002	862 (14.54)	4026 (9.83)	<0.0001
Hyperlipidemia, n (%)	94 (25.75)	19 (10.27)	<0.0001	1661 (28.02)	8888 (23.59)	<0.0001
Heart failure, n (%)	213 (58.36)	60 (32.43)	<0.0001	3221 (54.34)	14,120 (37.47)	<0.0001

BMI—body mass index; SBP—systolic blood pressure; DBP—diastolic blood pressure; AS dpmax—antegrade gradient across the narrowed aortic valve; EF LV—left ventricular ejection fraction, AR—aortic regurgitation; COPD—chronic obstructive pulmonary disease; CAD—coronary artery disease.

**Table 3:** Performance evaluation with comorbidities.

	Precision	Recall	F1 Score	Accuracy	AUC
ANN	**0.83**	0.72	0.77	0.81	0.78
SVM	0.77	0.78	0.78	0.80	0.79
Decision Tree	0.79	0.81	0.78	0.82	0.79
Random Forest	0.79	0.81	0.80	0.83	0.80

## Data Availability

The datasets generated and analyzed for this study can be requested from the corresponding author.
